# Protecting and rescuing the effectors: roles of differentiation and survival in the control of memory T cell development

**DOI:** 10.3389/fimmu.2012.00404

**Published:** 2013-01-23

**Authors:** Sema Kurtulus, Pulak Tripathi, David A. Hildeman

**Affiliations:** Division of Cellular and Molecular Immunology, Department of Pediatrics, Cincinnati Children’s Hospital Medical Center, University of CincinnatiCincinnati, OH, USA

**Keywords:** CD8^+^ T cells, memory cells, KLRG1^hi^CD127^lo^, Bim, Bcl-2

## Abstract

Vaccines, arguably the single most important intervention in improving human health, have exploited the phenomenon of immunological memory. The elicitation of memory T cells is often an essential part of successful long-lived protective immunity. Our understanding of T cell memory has been greatly aided by the development of TCR Tg mice and MHC tetrameric staining reagents that have allowed the precise tracking of antigen-specific T cell responses. Indeed, following acute infection or immunization, naïve T cells undergo a massive expansion culminating in the generation of a robust effector T cell population. This peak effector response is relatively short-lived and, while most effector T cells die by apoptosis, some remain and develop into memory cells. Although the molecular mechanisms underlying this cell fate decision remain incompletely defined, substantial progress has been made, particularly with regards to CD8^+^ T cells. For example, the effector CD8^+^ T cells generated during a response are heterogeneous, consisting of cells with more or less potential to develop into full-fledged memory cells. Development of CD8^+^ T cell memory is regulated by the transcriptional programs that control the differentiation and survival of effector T cells. While the type of antigenic stimulation and level of inflammation control effector CD8^+^ T cell differentiation, availability of cytokines and their ability to control expression and function of Bcl-2 family members governs their survival. These distinct differentiation and survival programs may allow for finer therapeutic intervention to control both the quality and quantity of CD8^+^ T cell memory. Effector to memory transition of CD4^+^ T cells is less well characterized than CD8^+^ T cells, emerging details will be discussed. This review will focus on the recent progress made in our understanding of the mechanisms underlying the development of T cell memory with an emphasis on factors controlling survival of effector T cells.

## IMMUNOLOGICAL MEMORY

The concept of immunological memory has dated back to as early as the fifth century B.C. as the Athenian author Thucydides mentioned in his scripts that people who survived plague would not be attacked a second time (Thucydides and Marchant, 1899). In seventh century, people drank snake venoms to get toxoid-like immunity (Plotkin et al., 2008). In ancient China, people blew powdered scabs of smallpox pustules into their nose to be protected from smallpox, a process called variolation (Plotkin et al., 2008). The process of variolation transferred to westward to the Middle East along shipping routes when Lady Mary Wortley Montagu witnessed this process and popularized variolation in England in the 1700s. By the time Edward Jenner immunized a child with cowpox and challenged him with smallpox, the concept of immune “memory” or “immunity” existed. Nearly 100 years elapsed before purposeful development of vaccines was attempted against cholera toxin and the rabies virus by Pasteur (Plotkin et al., 2008). Thus, the concept that prior exposure to a disease-causing microorganism (or a close relative) could provide long-lasted protection against subsequent infection has been around for a very long time. The subsequent large-scale development of effective vaccines against yellow fever, smallpox, rabies, influenza, polio, measles, mumps, diphtheria, Bordetella, hepatitis B, and, more recently, rotavirus have saved countless lives and are one of the greatest improvements to human health. Over the last few decades with the advent of cellular and molecular approaches we are started to unravel the mechanisms underlying immunological memory.

Immunological memory has been defined simply as the heightened immune response against a previously encountered pathogen that is due to the increased numbers of antigen-specific cells and their increased capacity to respond to secondary stimulation (Murphy et al., 2011). Both arms of adaptive immunity; antibody responses and T cell responses are quantitatively and qualitatively better than the primary responses. Immunological memory has been utilized successfully for generating protective immunity against many pathogens (Rappuoli, 2007). While it is clear that B cell production of antibody is critical for the protective features of many vaccines; long-lived T cell immunity is also critical component induced by vaccines. This review will focus on recent advances made in our understanding of mechanisms underlying the development of memory T cell responses.

## TRACKING T CELL RESPONSES

One of the substantial developments in T cell biology over the past few decades has been the ability to monitor T cells responses at the single-cell level. Early work examining T cell function was restricted to population based assays such as proliferation (^3^H incorporation) for CD4^+^ T cells and CTL assays (^51^Cr release) for CD8^+^ T cells. The development of TCR Tg mice and adoptive transfer approaches for the first time allowed tracking antigen-specific (albeit monoclonal) T cell responses to nominal antigens like ovalbumin (Kearney et al., 1994), to autoantigens (Katz et al., 1993), or to viral antigens (Pircher et al., 1990). It was not until the development of intracellular cytokine analysis by flow cytometry, that endogenous, polyclonal, antigen-specific T cell responses could be tracked at the single-cell level (Jung et al., 1993). While this was a critical development, it also required a brief stimulation of T cells either *in vitro* (Jung et al., 1993) or *in vivo* (Liu and Whitton, 2005), which could change the gene expression and phenotype of the cells. In addition, it only allowed for examination of cells whose cytokines are being measured, not necessarily all of the T cells responding to the antigen/infection. In contrast, the development of MHC tetramers was an absolutely critical tool for the tracking and analysis of endogenous T cell responses without the need for secondary stimulation (Altman et al., 1996). The development of these tools for tracking endogenous T cell responses has taught us a lot about T cell expansion, differentiation, and localization.

## KINETICS OF T CELL RESPONSES

**FIGURE 1:**
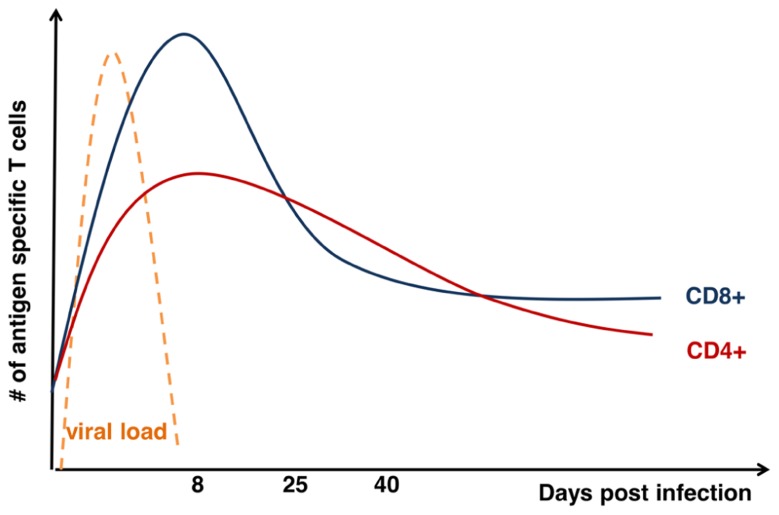
**Kinetics of T cell response after acute viral infection**. Graph shows total numbers (*y*-axis) of antigen-specific CD8^+^ (blue) and CD4^+^ (red) T cells days (*x*-axis) after acute infection as modified from Hinds et al. (2007). Kinetics of viral load (orange) is also displayed on the graph.

The initial reports tracking endogenous T cell responses characterized a massive expansion phase, in which responding T cells undergo 15–20 rounds of division, a “contraction” phase in which 80–90% of the responding T cells undergo apoptosis, and a “maintenance” phase in which the remaining effector cells persist as memory T cells and are maintained for the life of the animal (Butz and Bevan, 1998; Murali-Krishna et al., 1998; Williams and Bevan, 2007). For acute infections, the decline of T cell responses occurs just after the infection is cleared (**Figure 1**). Further, the expansion and contraction of CD8^+^ T cell responses are of a significantly greater magnitude compared with CD4^+^ T cell responses (**Figure 1**). While CD8^+^ T cell memory appears relatively stable over time, the CD4^+^ memory T cell population undergoes a gradual attrition (**Figure 1**). Nonetheless, a central question regarding the development of T cell memory is how some T cells avoid death and develop into memory T cells. Over the last decade, significant progress has been made regarding our understanding of the molecular mechanisms that contribute to the death of most effector T cells and to the transcriptional network that controls development of cells that are destined to become memory T cells. Herein, we will describe the current understanding of how T cells transit from potent effectors to lifelong protectors.

## HETEROGENEITY OF EFFECTOR T CELLS

Effector CD8^+^ T cells are a heterogeneous population as defined by differential expression of surface markers. As only a small fraction of effector T cells develop into memory cells, there has been a quest to identify memory precursors early after infection. Initially, it was unclear if memory cells went through an effector stage or whether they were a distinct lineage without effector characteristics. By using IFN-γ reporter mice or granzyme B promoters, it has been shown that memory CD8^+^ T cells were derived from IFN-γ producing and granzyme expressing effector cells, respectively (Harrington et al., 2008; Bannard et al., 2009). Importantly, adoptive transfer of a single naïve TCR transgenic T cell into congenic mice generated heterogeneous subsets of effector and memory CD8^+^ T cells in response to *L. monocytogenes* (Stemberger et al., 2007). Although these studies showed that memory cells are derived from effector cells, not every effector cell maintains the same potential to become memory cell over the course of infection. Many markers including cytokine receptors, chemokine receptors, and stimulatory/inhibitory receptors (described in more detail below) have been found to be differentially expressed among effector cells at the peak of the response (days 8–10 after infection). Among these markers, IL-7Rα (CD127) which is down-regulated on most of the effector cells early after infection (Schluns et al., 2000), but the proportion of cells expressing CD127 increases as the response contracts (Kaech et al., 2003).

Further characterization of these effector CD8^+^ T cells has revealed inverse expression of another marker, killer cell lectin-like receptor subfamily G, member 1 (KLRG1) relative to expression of CD127 (Joshi et al., 2007). At the peak of the response, two major CD8^+^ effector T cell populations emerge, one being KLRG1^hi^CD127^lo^ and another being KLRG1^lo^CD127^hi^ (Joshi et al., 2007). Although the two subsets had similar cytotoxicity and IFN-γ production, KLRG1^lo^CD127^hi^ cells had better production of IL-2 (Sarkar et al., 2008). Adoptive transfer experiments using TCR Tg cells have revealed that KLRG1^hi^CD127^lo^ cells slowly declined over time after transfer while KLRG1^lo^CD127^hi^ cells were maintained at a greater level and persisted as long-lived memory T cells (Joshi et al., 2007; Sarkar et al., 2008). Because of these results, KLRG1^hi^CD127^lo^ have been referred to as “short-lived effector cells or SLECs” and KLRG1^lo^CD127^hi^ have been referred to as “memory precursor effector cells or MPECs” (Joshi et al., 2007). While these markers have been helpful in identifying certain populations of cells that have enrichments of cells with more or less potential to develop into memory, further work is necessary to more precisely define cells with memory potential. For example, while many SLECs die during contraction of the response, not all do, and after contraction of the response is largely complete, roughly half of the CD8^+^ T cells have an SLEC phenotype (Joshi et al., 2007; Kaech and Wherry, 2007). Likewise, when assessing the numbers temporally, roughly 30–40% of MPECs die during contraction of the response (Sarkar et al., 2008; Kurtulus et al., 2011).

Other markers in addition to KLRG1 and CD127 are also used to determine the memory potential of effector T cells. For instance, CD127^hi^ cells also express high levels of CD27, which is a member of tumor necrosis factor receptor (TNF-R) family and the chemokine receptor, CXCR3, but these cells are found to have low expression of CD43 after infection with the Sendai virus (Kaech et al., 2003; Hikono et al., 2007). Thus, while these markers have helped identify cells with more or less ability to form long-lived memory cells, further work is necessary to more precisely define the cells within these subsets.

As primary infections have been difficult to assess in humans, it is unclear whether or not these precise effector subsets exist amongst human T cells. However, recent studies have shed light on effector cells in humans after vaccination with yellow fever virus and the smallpox vaccine (Miller et al., 2008; Akondy et al., 2009). Using MHC–peptide tetramers, the authors characterized the antigen-specific T cell response across the effector response and into memory from the peripheral blood. The phenotype of activated CD8^+^ T cells were characterized by high expression of HLA-DR and CD38 along with high expression of the proliferation marker Ki-67 and low expression of anti-apoptotic protein Bcl-2 and CD127 (Miller et al., 2008). As antigen-specific human T cells progressed into memory, they upregulated expression of CD127, CCR7, CD45RA, CD28, and Bcl-2 (Miller et al., 2008; Akondy et al., 2009). These memory cells were poly-functional and maintained after 2 years (Akondy et al., 2009). Although KLRG1 expression was not assessed in these studies, they showed that CD127 expression was similar in human and mouse T cells after infection. Thus, these studies showed that human and mouse effector CD8^+^ T cells share similar expression of several markers.

A more recent study performed a comprehensive analysis of 17 cell surface markers and 9 functional qualities of human CD8^+^ T cell subsets using single-cell spectrometric analysis (cytometry by time-of-flight or CyTOF; Newell et al., 2012). Functional qualities including expression of six different cytokines and cytotoxic granule components granzyme B and perforin were examined together with surface markers including CD62L, CD45RA, CD45RO, CD27, CD43, and KLRG1. This study found that naïve cells (CD45RA^+^ CD27^+^ CD62L^+^ CCR7^+^), central memory CD8^+^ T cells (T_CM_; CD45RA^-^ CD27^+^ CD62L^+^ CCR7^+^), effector memory CD8^+^ T cells (T_EM_; CD45RA^-^ CD27^-^ CD62L^-^ CCR7^-^) cells and terminal effector cells (CD45RA^+^ CD27^-^ CD62L^-^ CD28^-^ KLRG1^+^ CD57^+^) represented quite distinct subsets as previously described (Sallusto et al., 1999). However, this study also found a range of cells with combinatorial diversity of phenotypic and functional markers in between these subsets suggesting a continuum of T cell phenotypes (Newell et al., 2012). Unfortunately, this did not longitudinally assess the response to infection as samples were obtained from chronically infected individuals. Thus, more work is needed to temporally examine the effector T cell subsets in humans during both acute and chronic infections in greater detail.

## GENERATION OF EFFECTOR CD8^+^ T CELL SUBSETS

Recent work from a few labs has examined the potential *in vivo* plasticity of these subsets and has tracked their emergence from their naïve precursors. Interestingly, at the earliest times after the response when the cells can be reliably detected, a population appears that is KLRG1^lo^CD127^lo^, which have been termed “early effector cells or EECs” (Obar et al., 2010). When EECs were adoptively transferred into timed-infected recipient mice, they were able to generate both KLRG1^lo^CD127^hi^ and KLRG1^hi^CD127^lo^ cells; transferred KLRG1^lo^CD127^hi^ cells were able to give rise to some EECs early after transfer but predominantly remained as KLRG1^lo^CD127^hi^; while transferred KLRG1^hi^CD127^lo^ cells were largely unable to generate KLRG1^lo^CD127^hi^ cells (Obar et al., 2011). Thus, shortly after the response, naïve T cells lose expression of CD127, some cells stably reacquire CD127 expression, while others upregulate KLRG1 and largely fail to upregulate CD127 (Joshi et al., 2007; Sarkar et al., 2008). At a molecular level this regulation of CD127 appears to be due to the competing effects of Gfi-1 and GABP-α at the CD127 locus (Chandele et al., 2008). However, the mechanism(s) that control expression of Gfi-1 and GABP-α remain unclear.

As differential expression of KLRG1 and CD127 has allowed some demarcation of cells with more or less memory potential, much work has been focused on mechanisms underlying their generation. For example, one critical question is whether CD127 or KLRG1 are involved in the fate of effector T cells or whether they are simply markers. One initial idea was that expression of CD127 allowed effector CD8^+^ T cells to compete for IL-7 and, in doing so, was instructive for their survival and/or development into memory cells. However, while exogenous IL-7 could protect effector CD4^+^ and CD8^+^ T cells from contraction of the response (Tripathi et al., 2007; Nanjappa et al., 2008), transgenic expression of CD127 failed to prevent contraction of the response (Hand et al., 2007; Haring et al., 2008). Similarly, neutralization or inhibition of IL-7 after infection failed to substantially exacerbate contraction of the effector CD4^+^ or CD8^+^ T cell responses (Klonowski et al., 2006; Tripathi et al., 2007, 2010). A recent study has shown that KLRG1-deficient mice have no defects in memory T cell development (Grundemann et al., 2010), demonstrating that KLRG1 is not necessary for effector/memory T cell differentiation. However, given that there are multiple KLRG family members and the fact that KLRG1 possesses an immunoreceptor tyrosine-based inhibition motif (ITIM)-domain, makes it possible that KLRG1 contributes redundantly with other KLRG family members to limit signaling events within KLRG1^hi^CD127^lo^ cells. On the other hand, if neither KLRG1 nor CD127 are instructive, what are the mechanisms that control generation of these two subsets?

## INFLAMMATION DIRECTS EXPANSION OF KLRG1^hi^CD127^lo^ CELLS

Recent work has revealed an intriguing and complex interrelationship between transcriptional programs that balance input from surrounding inflammatory stimuli to promote a self-renewal program that maintains lifelong immunity. The transcription factor t-bet, initially described as a master regulator of Th1 fate, favors the generation of KLRG1^hi^CD127^lo^ CD8^+^ T cells (Joshi et al., 2007). Loss of tbx21 (gene encoding t-bet) reduced the formation of KLRG1^hi^CD127^lo^ effector CD8^+^ T cells, while graded increases in t-bet expression, whether retrovirally overexpressed or induced by varying amounts of inflammatory stimuli (e.g., TLR stimuli, IL-12, etc.) gradually increased the generation of KLRG1^hi^CD127^lo^ CD8^+^ T cells (Badovinac and Harty, 2007; Joshi et al., 2007). Importantly, the overall numbers of KLRG1^lo^CD127^hi^ CD8^+^ T cells in these t-bet titration experiments did not change, suggesting a critical role of t-bet in the formation of cells with a KLRG1^hi^CD127^lo^ phenotype, but not an MPEC phenotype.

Importantly, KLRG1^hi^CD127^lo^ cells express more t-bet compared to KLRG1^lo^ CD127^hi^ cells, and the reverse is true for eomesodermin (eomes; Joshi et al., 2007, 2011). While neither subset is truly negative for either molecule, both are required for expression of CD122 and a lower t-bet:eomes ratio correlates with KLRG1^lo^CD127^hi^ cells and long-lived memory (Intlekofer et al., 2005, 2007; Banerjee et al., 2010). Thus, control of the t-bet:eomes ratio, as dictated by the level of inflammation is likely critical in controlling CD8^+^ T cell memory generation.

**FIGURE 2:**
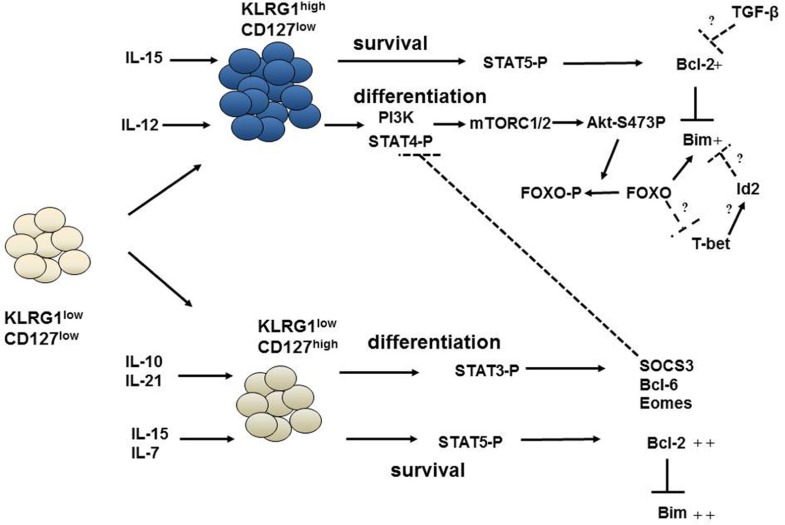
**Pathways governing the survival and differentiation of effector CD8^+^ T cells**. The differentiation of early effector cells (KLRG1^lo^CD127^lo^) into KRLG1^hi^CD127^lo^ or KLRG1^lo^CD127^hi^ cells is regulated by inflammatory cytokines and by IL-10 and IL-21. IL-12 can activate STAT4 and PI-3K signaling which modulates mTOR kinases and subsequent Akt phosphorylation at s473. Phosphorylated Akt can phosphorylate and inactivate FOXO proteins. This favors an increased t-bet:eomes ratio and differentiation into KLRG1^hi^CD127^lo^ cells. On the other hand, Stat3 phosphorylation by IL-10 and IL-21 increases eomes and other transcription factors required for differentiation of KLRG1^lo^CD127^hi^ cells. SOCS3 induced by Stat3 can, then, inhibit IL-12 signaling, effectively shielding KLRG1^lo^CD127^hi^ cells from inflammation. Interestingly, survival of effector subsets are regulated by γc cytokines IL-15 and IL-7 via signals driven through STAT5 which appear largely independent of differentiation. While KLRG1^hi^CD127^lo^ cells can only receive IL-15 signals; IL-15 and IL-7 can both activate Stat5 signaling in KLRG1^lo^CD127^hi^ cells. This results in Bcl-2 upregulation and inhibition of Bim-mediated apoptosis. IL-15 becomes limiting for KLRG1^hi^CD127^lo^ cells reducing their ability to sustain Bcl-2 levels in the face of TGF-β signaling. Id2 may also control Bim levels in KLRG1^hi^CD127^lo^ cells. FOXO proteins may be at the intersection of survival and differentiation pathways as it can regulate both Bim expression and influence the t-bet:eomes ratio.

Understanding the regulation of this t-bet:eomes balance is the focus of several recent papers, which have outlined a complex interplay between t-bet and the mTORC1/AKT/FOXO signaling network (**Figure 2**). Overexpression of a constitutively active (ca) AKT transgene led to significantly increased expression of t-bet and a concomitant decrease in eomes expression (Kim et al., 2012). Conversely, caFOXO overexpression decreases expression of t-bet and increases expression of eomes (Rao et al., 2012). Inflammation, via IL-12 (and possibly other inflammatory mediators) has been shown to increase mTORC1/AKT, which in turn decrease FOXO activity and enhance t-bet expression (Rao et al., 2010, 2012). However, a complicating factor in many of these studies is that caAKT appears to also decrease expression of CD127 (Hand et al., 2010), likely through inactivation of FOXO1 (Kerdiles et al., 2009), making it difficult to clearly distinguish the effector CD8^+^ subsets. Thus, while the current data suggest that mTORC1/AKT/FOXO signaling is differentially balanced between the subsets, it is also formally possible that a proper balance of mTORC1/AKT/FOXO signaling is necessary to emerge from the EEC compartment. More work will be necessary to cleanly dissect the factors that control mTORC1/AKT/FOXO signaling between the effector subsets.

## THE ROLE OF ANTIGEN PRESENTATION IN THE GENERATION OF EFFECTOR AND MEMORY CD8^+^ T CELLS

Obviously, antigen initially drives the metamorphoses of naïve to effector T cell. Previous studies showed that limiting antigen exposure to the first 24 h was sufficient to drive expansion and differentiation into full-fledged effector (van Stipdonk et al., 2001) and memory (Kaech and Ahmed, 2001) cells *in vitro*. However, stimulation longer than 40 h in the presence of IL-12 resulted in a substantial increase in CD8^+^ clonal expansion compared to shorter stimulation, indicating the role of inflammatory cytokines in the magnitude of the response (Curtsinger et al., 2003). These studies suggested at least two interactions of T cells with antigen-presenting cells (APCs) promoted optimal effector and memory responses. Interestingly, limiting antigen display during *Listeria* infection by antibiotic treatment 24 h after infection resulted in a decreased magnitude of the response but a similar contraction (Badovinac et al., 2002). Secondary challenge of the antibiotic treated mice revealed an enhanced secondary response, despite the decreased magnitude of the primary response (Badovinac et al., 2002). Subsequently, it was found that antibiotic treatment prior to infection in this same model resulted in significantly enhanced generation of cells with a memory phenotype (CD127^hi^; Badovinac et al., 2004). However, in this study, it was shown that antibiotic treatment significantly decreased inflammation, and it was this attribute, rather than effects on antigen display that likely contributed to the increased memory cells (Badovinac et al., 2004). Similarly, adoptive transfer of naïve TCR Tg cells into mice with an ongoing immune response (as inflammation is waning), results in accelerated development of memory cells (D’Souza and Hedrick, 2006; Sarkar et al., 2007). Furthermore, attempts to restrict antigen display by elimination of dendritic cells (DCs) using CD11c-DTR mice also resulted in a decreased magnitude of the CD8^+^ T cell response, but accelerated development of cells with memory characteristics (Prlic et al., 2006). However, as DCs are also the same cells that secrete pro-inflammatory mediators, the degree to which their role as antigen presenters versus producers of inflammation is difficult to separate. Thus, while it is likely that limiting antigen display may contribute to memory cell development, further work is necessary to cleanly separate inflammatory stimuli from antigen-presentation.

## MIGRATION AND LOCALIZATION OF EFFECTOR AND MEMORY CD8^+^ T CELL SUBSETS

Being at the right place at the right time may also be important for memory cell development. Indeed, recent work has shown that, in the spleen KLRG1^lo^CD127^hi^ CD8^+^ T cells are mostly localized to the T cell zones in the white pulp, while KLRG1^hi^CD127^lo^ CD8^+^ T cells are localized to the red pulp (Jung et al., 2010). CXCR3 signals may also be critical in attracting KLRG1^hi^CD127^lo^ cells to the marginal zone areas, where they may be exposed to more inflammatory stimuli (Kurachi et al., 2011). Also, T cells in CXCR3/CCR5-deficient mice had similar problems with localization, actually failed to undergo contraction in the spleen, and had an emergence of KLRG1^lo^CD127^hi^ cells (Kohlmeier et al., 2011). In these studies, it was also clear that the absence of CXCR3/CCR5 restricted the accumulation of effector T cells to sites of infection/inflammation as plenty of cells were recruited to the lung, but not to areas of viral replication within the infected lung. While overall tissue localization may direct CD8^+^ T cells to general areas of inflammation, the finer tuning of their migration within these organs is likely mediated by signals through CXCR3/CCR5. Intriguingly, CXCR3 signals through AKT/FOXO transcription factors raising the intriguing possibility that, in addition to promoting appropriate localization, differentiation signals driven by these chemokine receptors may also contribute to effector T cell heterogeneity. Conversely, high expression of CCR7 on KLRG1^lo^CD127^hi^ cells likely fosters their migration to/retention within the T cell zones where the ligands CCL19 and CCL21 are highly expressed (Jung et al., 2010). This differential expression of CCR7 may be part of the effector T cell transcriptional program as t-bet and B lymphocyte-induced maturation protein-1 (Blimp-1) can suppress CCR7 expression. Further, in the lymph nodes, fibroblastic reticular cells in T cell zones produce CCL19 and IL-7 (Link et al., 2007), thereby linking localization to T cell zones by CCR7 to IL-7 signals supporting survival of effector cells. It will be of great interest to determine whether it is simply the localization driven by chemokines that is critical for effector CD8^+^ T cell differentiation, or whether signaling by these chemokine receptors also contributes to effector cell heterogeneity and memory development.

## TRANSCRIPTIONAL PROGRAMING OF KLRG1^hi^CD127^lo^ EFFECTOR T CELLS

In addition to t-bet, other transcription factors have been shown to contribute to the formation of KLRG1^hi^CD127^lo^ cells including the inhibitor of differentiation 2 (Id2) and Blimp-1 (Kallies et al., 2009; Yang et al., 2011). Id family proteins act as transcriptional repressors and often combat e-box proteins (Murre, 2005). Of the four members of Id family, both Id2 and Id3 are reciprocally expressed in effector CD8^+^ T cell subsets. Id2 is more expressed in KLRG1^hi^CD127^lo^ cells, while Id3 is more expressed in KLRG1^lo^CD127^hi^ cells (Yang et al., 2011). Id2-deficient mice generated a substantially reduced effector CD8^+^ T cell response to *L. monocytogenes* (Cannarile et al., 2006). This was further characterized in a follow-up study, where Id2 was found to be required for formation of KLRG1^hi^CD127^lo^ cells; and Id3 was required for formation of KLRG1^lo^CD127^hi^ effector CD8^+^ T cells (Yang et al., 2011). Further studies showed that E proteins, E2A and HEB were required for generation of memory precursor KLRG1^lo^CD127^hi^ effector CD8^+^ T cells (D’Cruz et al., 2012). The limitation of E2A/HEB activity by Id proteins appears to set the balance between these two important effector T cell subsets.

Blimp1 is a transcription repressor in the PRDI-BF1 and RIZ homology domain containing (PRDM) family and also appears to contribute to formation of KLRG1^hi^CD127^lo^ cells (Kallies et al., 2009; Rutishauser et al., 2009). Similar to Id proteins and E-box proteins, Blimp1 and another transcription repressor in the BTB/PZ family, Bcl-6 act as antagonists of each other (Tunyaplin et al., 2004; Cimmino et al., 2008). Blimp1 expression is higher in KLRG1^hi^CD127^lo^ cells and the absence of Blimp-1 impairs development of these cells (Rutishauser et al., 2009). Multiple mechanisms may contribute to Blimp-1’s role in promoting KLRG1^hi^CD127^lo^ cells, including antagonization of Bcl-6 (Martins et al., 2006; Kallies et al., 2009), repression of IL-2 production (Martins et al., 2008). A recent report suggests that Blimp-1 may repress expression of Id3 in KLRG1^hi^CD127^lo^ cells and that lack of this repression (i.e., in Blimp-1-deficient mice) allows for their persistence into the memory compartment and for expression of E2A-driven genes important for genomic stability (Ji et al., 2011). Thus, the current data suggest a model in which inflammation drives expression of t-bet and an AKT/mTOR/FOXO signaling network that may contribute directly (by inducing Id2/Id3) or potentially in parallel with a Bcl-6/Id2/Id3 repressive network.

## TRANSCRIPTIONAL PROGRAMING OF MEMORY PRECURSOR EFFECTOR T CELLS

Memory precursor effector cells are the Yin to the SLEC Yang and as such are often intertwined, experimental interpretations notwithstanding. Nonetheless, several factors have been reported to control the development of this effector cell population, including Bcl-6, TCF-1, and Stat3 (**Figure 2**). Deficiency in Tcf-1, an effector of the Wnt signaling pathway, impairs proliferative responses against *Listeria* infection and generation of KLRG1^lo^CD127^hi^ effector CD8^+^ T cells after *Listeria* (Zhou et al., 2010) and lymphocytic choriomeningitis virus (LCMV) infections (Jeannet et al., 2010). Zhou et al. (2010) also showed that Tcf-1 is essential for optimal eomes and IL-2Rβ expression and forced overexpression of eomes partially prevented the decline of effector cells, although it did not appear to affect their surface marker phenotype. However, a role for β-catenin/wnt signaling on memory generation is controversial as two recent studies found that loss of β-catenin did not impair generation of effector responses (Driessens et al., 2010; Prlic and Bevan, 2011). In these studies, T cell-specific loss of β-catenin did not impair effector or secondary responses (as assessed by the frequency of tetramer^+^ T cells up to day 30 after infection); however, the expression of KLRG1/CD127 markers were not assessed in this study (Prlic and Bevan, 2011). Although it is possible that a β-catenin-independent function of Tcf-1 could contribute to formation of KLRG1^lo^CD127^hi^ cells, at least one study suggests that the effects of Tcf-1 on memory T cell development require its ability to interact with β-catenin (Jeannet et al., 2010). Thus, more work is required to determine the role of the wnt/β-catenin/Tcf-1 pathway on KLRG1^lo^CD127^hi^ cell formation and memory development.

Another recent study implicated STAT3, downstream of IL-10 and IL-21 signaling as a critical regulator of development of memory precursor cells (Cui et al., 2011). Interestingly, this study showed that T cell-specific loss of STAT3 or neutralization of IL-10 in an IL-21-deficient background lead to decreased percentage and number of KLRG1^lo^CD127^hi^ cells but an increased number of KLRG1^hi^CD127^lo^ cells (Cui et al., 2011). Thus, while the overall numbers of effector cells did not change, their phenotype did, an important distinction and potential separation of the effects of differentiation from effects on survival at a time when responses are crashing. In this study, Stat3-deficient effector T cells had normal expression of eomes, Blimp-1, and Bcl-6 at the peak of the response their levels decreased over time (Cui et al., 2011). However, it was not apparent if this was a selective decrease in KLRG1^lo^CD127^hi^ cells or the decrease was reflective of a shift in the effector subpopulations (Cui et al., 2011). SOCS-3, a known STAT-3 target gene was increased in wild type (WT) KLRG1^lo^CD127^hi^ cells at the peak of the response, and these levels were decreased in STAT-3-deficient cells, but again, subset-specific expression was not clear. Nonetheless, SOCS-3 overexpression in effector T cells reduced their ability to activate STAT4, whilst SOCS-3 knockdown promoted emergence of KLRG1^hi^CD127^lo^ cells. However, it remains unclear as to how these target genes may be selectively activated in KLRG1^lo^CD127^hi^ cells because stimulation of effector CD8^+^ T cells with IL-10 and IL-21 lead to homogenous STAT3 activation (Cui et al., 2011). Together, the data suggest an intriguing model whereby KLRG1^lo^CD127^hi^ cells are shielded from the differentiating effects of inflammation by STAT3-driven induction of SOCS-3.

## PARALLELS BETWEEN EFFECTOR AND MEMORY SUBSETS

First described in humans, T_CM_ express lymph node homing receptors CD62L and CCR7 and are mostly found in the lymph nodes and spleen as opposed to the T_EM_ that lack CD62L and CCR7 expression and instead express a variety of chemokine receptors and tissue-specific homing receptors (Sallusto et al., 1999; Masopust et al., 2001). These two subsets also differ in their functional properties. T_CM_ cells are capable of IL-2 production, self-renewal and they are multi-potent cells that can rapidly proliferate upon activation and generate effector cells (Wherry et al., 2003). Numbers of T_CM_ cells gradually increase over time and outnumber T_EM_ cells. While some studies suggest that the T_EM_ subset converts to T_CM_ over time (Wherry et al., 2003), others suggest that these lineages branch out early during memory differentiation and they are not convertible (Marzo et al., 2005). Adoptively transferred T_EM_ cells were able to convert to CD62L^hi^, CCR7^hi^ CD27^hi^ cells that could produce IL-2 (Wherry et al., 2003). However, responses of non-physiologically high numbers of P14 TCR transgenic T cells were shown to be different qualitatively compared to endogenous effector cells (Marzo et al., 2005). Nevertheless, both endogenous T_EM_ cells and transfers of low number of P14 cells were shown to convert to T_CM_ subset upon transfer (Sarkar et al., 2007). Although the conversion contributes to the increase in T_CM_ numbers, CD62L^hi^ effector T cells can be detected early after the infection and they are enriched within the KLRG1^lo^CD127^hi^ subset in the lymph nodes (Obar et al., 2011). On the other hand, KLRG1^hi^CD127^lo^ cells are low for the expression of CD62L (Sarkar et al., 2008). Thus, in addition to conversion, higher proliferation and better survival of T_CM_ cells also contributes to the outgrowth of T_CM_ cells later, after the infection has cleared. Reacquisition of T_CM_ phenotype can be much slower after prime-boost immunizations (Jabbari and Harty, 2006; Masopust et al., 2006). Also, a greater fraction of secondary memory cells are KLRG1^hi^CD127^hi^CXCR3^lo^CD27^lo^ phenotype (Masopust et al., 2006; Joshi et al., 2011). Although the recall responses of the adoptively transferred secondary memory cells were found to be even more potent than the responses of primary memory cells (Jabbari and Harty, 2006; Masopust et al., 2006), third generation memory cells had lower recall responses upon adoptive transfers as a result of further differentiation into KLRG1^hi^ phenotype (Masopust et al., 2006). However, if the prime-boost immunizations are done in the same host; increased numbers of pre-existing memory cells prevent further differentiation into KLRG1^hi^CXCR3^lo^CD62L^lo^CD27^lo^ phenotype (Joshi et al., 2011). Thus, the numbers of memory cells generated and the context of secondary priming conditions may affect the phenotype of secondary memory cells and these differences could play a role in the efficacy of prime-boost immunizations.

There are different models to explain the differentiation of memory cells from effector cells:

The early fate determination model predicts that memory cell heterogeneity, CD62L^hi^ – CD62L^lo^ or CD127^hi^ – CD127^lo^ are fixed (pre-determined) at early times after infection. Indeed, effector cells with CD62L expression (Obar and Lefrancois, 2010) or CD127 expression (Kaech et al., 2003) can be detected before the peak of immune response. Similarly, Chang et al. (2007) visualized the TCR transgenic cells after priming and just before the first division and found that certain cell surface molecules or TCR signaling components segregated asymmetrically during division. They showed that certain receptors segregated to the putative distal pole relative to the microtubule organizing center (MTOC) which is formed close to the immunological synapse. This resulted in asymmetric cell division and the daughter cell containing the distal pole as to the synapse had more characteristics of memory T cells such as CD62L. This study, although incomplete, provided a mechanism as to how heterogeneity can be generated from a single CD8^+^ T cell during the first division (Chang et al., 2007). However, CD62L^lo^ effector cells can also convert to CD62L^hi^ cells (Wherry et al., 2003; Sarkar et al., 2007) which suggest that there is some flexibility during memory differentiation.

The decreasing potential model suggests that every effector cell has the potential to develop into a memory cell, but exposure to inflammation and antigen for longer periods of time can further differentiate effector cells into terminal effector cells (KLRG1^hi^CD127^lo^) and decrease their potential to become memory cells (Ahmed and Gray, 1996; Badovinac et al., 2005; D’Souza and Hedrick, 2006).

A modified model proposed by Kaech and Wherry (2007), fate commitment with progressive differentiation suggests that there are memory precursors within the KLRG1^lo^CD127^hi^ generated early in the immune response, but these cells are not fully mature memory cells and they require further differentiation. Although these cells have the potential for memory differentiation, they can develop into terminal effector cells (KLRG1^hi^CD127^lo^) if they encounter inflammatory signals. Importantly, this model appears to be consistent for the host response to several diverse infections (D’Souza and Hedrick, 2006; Badovinac and Harty, 2007; Joshi et al., 2007). As mentioned previously, IL-10 and IL-21 act to “shield” KLRG1^lo^CD127^hi^ cells from the effects of inflammation, by increasing expression of SOCS3, which limits STAT-driven signals from inflammatory receptors (Cui et al., 2011). Thus, while differential expression of KLRG1 and CD127 can crudely mark cells with more or less memory potential, they likely require additional maturation signals and shielding from pro-inflammatory cytokines as they develop into full-fledged memory T cells.

## ENDOGENOUS MEMORY CELLS – IRRELEVANT BYSTANDER OR ACTIVE PARTICIPANT?

In addition to infection-induced memory cells, it is well known that mice harbor populations of pre-existing memory T cells that bear markers of memory (CD44, Ly6c, etc.). Notably, these cells arise in mice that have not been purposefully challenged with infection. Admittedly, some of these cells might be specific for infections existing in some mouse colonies, for environmental antigens, or for gut flora. However, endogenous memory cells exist in gnotobiotic mice and recent data suggest that a fair number of these cells arise during thymic development (Dobber et al., 1992; Weinreich et al., 2010). A complex cellular and cytokine network, involving NKT cells and IL-4 appears to contribute to the development of pre-existing memory T cells, at least in Balb/c mice (Weinreich et al., 2010). Interestingly, in the process of quantifying the pre-existing naïve T cell compartment in unchallenged animals using peptide–MHC tetramers, Kedl’s group found a significant frequency of T cells isolated from unchallenged mice bore memory markers (Haluszczak et al., 2009). They showed that, after purification these endogenous memory cells responded more robustly to stimulation, raising the intriguing possibility that this heterogeneity in the naïve compartment might contribute to effector T cell heterogeneity. On the other hand, in other experiments, transfer of a single TCR Tg T cell shows that effector and memory populations can arise from a single cell, a demonstration that differentiation from a common precursor is sufficient for effector and memory development. Whether or not there is a significant contribution of endogenous memory T cells to effector heterogeneity or whether these pre-existing cells contribute to epitope dominance at the population level (or both) remains to be determined.

## APOPTOSIS AND THE DEVELOPMENT OF T CELL MEMORY

The molecular mechanisms responsible for apoptotic cell death have been investigated intensely over the last few decades. Mammalian cells have two major pathways to execute apoptosis: the extrinsic pathway (activated by death receptors of the TNF-R superfamily); and the intrinsic pathway (mostly controlled by members of the Bcl-2 gene family; Strasser, 2005). A considerable amount of experimental effort has been put into understanding T cell apoptosis. Initially, based on the discovery that defects in Fas signaling led to the accumulation of T cells in autoimmune lymphoproliferative syndrome (ALPS) patients and lpr/gld mice, and the requirement for Fas in an *in vitro* model of activated T cell death it was assumed that Fas signaling was required for the contraction of T cell responses (Watanabe-Fukunaga et al., 1992; Takahashi et al., 1994; Brunner et al., 1995; Dhein et al., 1995; Fisher et al., 1995; Rieux-Laucat et al., 1995). However, while *in vitro* experiments readily showed a role for Fas/FasL signaling in activated T cell death, several experiments showed that contraction of T cell responses occurred readily *in vivo* in the absence of Fas signaling (Desbarats et al., 1998; Hildeman et al., 2002; Pellegrini et al., 2003). Thus, although disruptions of either pathway can affect T cell homeostasis, recent research has suggested a critical role for Bcl-2 family members and the intrinsic pathway in controlling contraction of T cell responses (Hildeman et al., 2002; Pellegrini et al., 2003; Wojciechowski et al., 2006).

The Bcl-2 family can be classified into three subfamilies that have either pro- or anti-apoptotic function. Group 1 consists of anti-apoptotic Bcl-2-like molecules that contain most or all of the four Bcl-2 homology (BH) domains. Group 2 consists of Bax-like molecules that are pro-apoptotic and contain BH domains 1–3. Group 3 consists of BH3-only molecules that are pro-apoptotic and whose only homology to Bcl-2 lies in a short 9–10 amino acid stretch termed the BH3 domain. Group 3 has the most members, which appear to be expressed in a relatively tissue-specific fashion (Youle and Strasser, 2008). BH3-only molecules appear to transmit apoptotic signals to group two Bax-like molecules. In the absence of the two predominant Bax-like molecules, Bax and Bak, BH3-only proteins fail to induce apoptosis (Zong et al., 2001). The mechanism(s) by which BH3-only molecules transmit signals to Bax-like molecules remains the subject of some controversy. One model proposes direct interactions between certain BH3-only molecules and Bax-like molecules (Letai et al., 2002; Kuwana et al., 2005; Kim et al., 2006), while another proposes that BH3-only molecules sequester anti-apoptotic molecules from Bax-like molecules and there is no direct interaction between BH3-only and Bax-like molecules (Willis et al., 2007). Despite the controversy both of these models highlight the importance of physical interactions between Bcl-2 family members in cell death/survival decisions. Thus, a major control point for cell death lies in the regulation of the balance between the levels of pro- and anti-apoptotic molecules.

The first experiment implicating Bcl-2 family members in activated T cell death showed that overexpression of Bcl-2 was sufficient to prevent T cell deletion in response to staphylococcal enterotoxin B (SEB; Strasser et al., 1991). We repeated this experiment and found that, in contrast to loss of Fas and TNF-R signaling, Bcl-2 overexpression gave a substantial protection from SEB-induced deletion (Hildeman et al., 2002). Likewise, loss of Bim provided a similar protection from deletion (Hildeman et al., 2002). Although previous reports showed that transgenic Bcl-2 overexpression failed to prevent contraction of viral-specific T cell responses, the level of the transgene across the response was never examined (Petschner et al., 1998). Notably, by mechanisms that remain unclear, the expression of endogenous Bcl-2 in the human Bcl-2 transgenic mice that were used is substantially decreased, if not all together absent (Jorgensen et al., 2007). Further, following T cell activation, the levels of the Bcl-2 transgene also decline, making it less potent. Subsequently, our and other groups have observed that the loss of the Bcl-2 antagonist, Bim, prevents contraction of antigen-specific CD4^+^ and CD8^+^ T cell responses to viral, bacterial, and parasitic infection (Pellegrini et al., 2003; Wojciechowski et al., 2006; Prlic and Bevan, 2008; Reckling et al., 2008). A major question is how T cells normally avoid Bim-driven death on their way to becoming memory T cells.

## REGULATION OF Bcl-2 BY γc CYTOKINES

Recent work from our and others groups have begun to address that question. In T cells, major controllers of Bcl-2 expression are the common gamma chain cytokines (Nakajima et al., 1997; Schluns et al., 2000; Berard et al., 2003; Wojciechowski et al., 2007). It has been known for some time that addition of IL-2, IL-4, IL-7, and IL-15 to activated or resting T cells promotes the expression of Bcl-2 (Vella et al., 1997, 1998) and Bcl-2 is largely required for *in vitro* T cell survival in response to these cytokines (Wojciechowski et al., 2007). The decreased expression of CD127 on the surface of KLRG1^hi^CD127^lo^ CD8^+^ T cells renders them less sensitive to IL-7 and largely dependent upon IL-15 (Joshi et al., 2007; Rubinstein et al., 2008; Tripathi et al., 2010). CD127^hi^ effector cells, on the other hand, require IL-7 and IL-15 for their optimal survival, although neutralization of IL-7 in an IL-15-deficient background only led to the loss of roughly half of this population (Tripathi et al., 2010). Thus, other γc cytokines probably also play a role in effector T cell survival because the loss of STAT5 signaling during the response led to a dramatic loss of both effector CD8^+^ T cell subsets (Tripathi et al., 2010). Mechanistically, STAT5 is critical for the ability of IL-7 and IL-15 to promote Bcl-2 expression (Tripathi et al., 2010). Thus, a common g cytokine/STAT5/Bcl-2 network is critical for maintaining effector CD8^+^ T cell responses (**Figure 2**).

While cytokine signaling through STAT5 promotes expression of Bcl-2 it has been reported that TGF-β signaling can antagonize Bcl-2 expression in KLRG1^hi^CD127^lo^ cells (Sanjabi et al., 2009). Adoptively transferred T cells expressing a dnTGF-βR transgene had substantially increased expansion of effector cells, which was accompanied by increased expression of Bcl-2 (Sanjabi et al., 2009). Further, there appeared to be an intersection with IL-15 in this model, as transfer of dnTGF-βR Tg T cells into IL-15 deficient mice led to a partial restoration of Bcl-2 levels compared to WT controls (Sanjabi et al., 2009). However, the increases in Bcl-2 were transient in dnTGF-βR Tg T cells and although there were increased T cells at the peak of the response, the contraction of the response was equal if not greater than the control, non-Tg animals (Sanjabi et al., 2009). Also, following T cell activation, levels of endogenous TGF-βR decline dramatically making it unclear if this pathway is operative during the normal response or whether it is magnified by transgenic dnTGF-βR overexpression.

## Bim/Bcl-2 BALANCE IN EFFECTOR CD8^+^ T CELL SUBSETS

Initial work describing KLRG1^lo^CD127^hi^ and KLRG1^hi^CD127^lo^ cells, showed that Bcl-2 expression was higher in KLRG1^lo^CD127^hi^ cells and this was attributed to their prolonged survival (Joshi et al., 2007; Sarkar et al., 2008), however this was not formally tested. Using a combination and genetic and pharmacologic approaches, we tested the role of Bcl-2 in effector T cell survival, and its role in combating Bim within the effector subsets. Interestingly, we found that while Bcl-2 levels were higher in KLRG1^lo^CD127^hi^ cells than KLRG1^hi^CD127^lo^ cells, that Bim levels were also higher (Kurtulus et al., 2011). Genetic loss or inhibition of Bcl-2 led to a massive, Bim-dependent loss of KLRG1^lo^CD127^hi^ cells, and a less profound, but still significant loss of KLRG1^hi^CD127^lo^ cells (Kurtulus et al., 2011). Notably, the cells that survived in the absence of Bcl-2 had significantly decreased levels of Bim (Kurtulus et al., 2011). This phenomenon may also explain the “Bcl-2 independence” of memory T cell survival reported in mice with a mutant IL-7Rα transgene that is incapable of activating STAT5 and maintaining significant levels of Bcl-2 (Osborne et al., 2007). Thus, it is likely that Bcl-2 is an obligate defender of Bim to maintain survival of the memory precursor population, although the additional loss of Bim did not completely restore precursor cell numbers, suggesting that, in addition to restraining Bim, Bcl-2 may antagonize other pro-apoptotic molecules. Nonetheless, these data showed that Bcl-2 levels determined the levels of Bim that effector T cells can tolerate and survive (**Figure 2**).

While decreased expression of Bcl-2 certainly contributes to the demise of effector CD8^+^ T cells, changes in Bim expression are difficult to detect because once past a certain Bcl-2 level, cells having higher expression of Bim would be lost by apoptosis. We circumvented this issue by using mice that were deficient in Bak, but had a T cell-specific loss of Bax, making them insensitive to death driven by BH3-only Bcl-2 family members (Zong et al., 2001; Kurtulus et al., 2011). Loss of Bax and Bak led to accrual of T cells with significantly increased levels of Bim, suggesting that there is indeed a rather significant transcriptional induction of Bim during the response (Kurtulus et al., 2011). Importantly, the levels of Bcl-2 were also decreased significantly in these “undead” cells effectively uncoupling concordant Bim and Bcl-2 expression. Because of the inherent toxicities associated with altered expression of Bim, it has been difficult to determine the factors that control Bim expression in T cells. However, recent work has suggested that FOXO3a and Id2 may be regulators of Bim within effector T cells, as loss of FOXO3a led to decreased Bim protein (Sullivan et al., 2012), while loss of Id2 led to increased Bim mRNA (Cannarile et al., 2006). It is possible that there are intersections between FOXO3a and Id2 proteins, as deficiencies in either molecule led to major effects on expansion but rather minor effects on contraction of the response (**Figure 2**). Thus, more work will be necessary to clearly examine the complex transcriptional network underlying effector T cell contraction.

## DEVELOPMENT OF CD4 MEMORY – CONSIDERABLY DIFFERENT FROM DEVELOPMENT OF CD8 MEMORY

Although much more work has been done to define effector T cell subsets and control of CD8^+^ T cell memory, clues are emerging to define effector CD4 responses and the development of memory CD4^+^ T cells. Interestingly, it appears that the markers expressed on effector CD8^+^ T cells and those expressed on effector CD4^+^ T cells are quite distinct. For example, expression of CD127 on effector CD4^+^ T cells is more dynamic; CD127^lo^ effector CD4^+^ T cells readily re-express CD127. Several recent studies have examined heterogeneity within effector CD4^+^ T cells. One study found that subsets of effector CD4^+^ T cells could be defined based expression of P-selectin ligand-1 (PSGL-1) and Ly6C into three distinct, PSGL-1^lo^^w^Ly6C^lo^^w^, PSGL-1^hi^Ly6C^lo^^w^, and PSGL-1^hi^Ly6C^hi^ (Marshall et al., 2011). Over time after infection, there was a slight enrichment for PSGL-1^hi^Ly6C^low^ cells, but this enrichment was not nearly as dramatic as the enrichment for KLRG1^lo^CD127^hi^ cells within the effector CD8 compartment. However, similar to the KLRG1^hi^CD127^lo^ CD8^+^ T cells PSGL-1^hi^Ly6C^hi^ CD4^+^ population required t-bet expression (Marshall et al., 2011). Thus, while effector PSGL-1^hi^Ly6C^low^ cells appeared to be more capable of expanding in response to a secondary challenge, and that they share a similar transcriptional profile with memory CD4^+^ T cells (Marshall et al., 2011), suggests that this subset most likely contains memory precursors.

On the other hand, another study defined effector CD4^+^ subsets via expression of CXCR5 and PD-1 (Pepper et al., 2011). Effector CD4^+^ T cells were again divided into three major subsets, cells expressing CCR7 along with intermediate levels of CXCR5 and lacking PD-1 (termed Tcm), those expressing t-bet, but not CCR7, CXCR5 nor PD-1 (Th1), and those expressing CXCR5 and PD-1 (Tfh). While Tfh cells waned dramatically over time, Th1 cells contracted more vigorously and Tcm cells contracted less vigorously (Pepper et al., 2011). In secondary responses, Tcm cells gave rise to all three subsets, while Th1 cells gave rise to only Th1 cells, suggesting that the Th1 cells, when they exist as memory cells are less able to give rise to the other subsets, while subset differentiation ability is maintained in the Tcm population. This study also showed that the Th1 cells largely required CD25 expression, while Bcl-6 was critical for Tcm cells as was inducible costimulator (ICOS) stimulation from B cells (Pepper et al., 2011). Importantly, while Tcm and Tfh both required Bcl-6, it is notable that they are discrete populations due to their differential localization after adoptive transfer and the fact that Tcm cells are inefficient at producing Tfh cells in secondary responses (Pepper et al., 2011). The overlap and relationship between the effector CD4^+^ T cell subsets identified by these two studies remains unclear and awaits further investigation.

The expansion and contraction of the CD4^+^ T cell response also shares both similarities and differences contraction of the CD8^+^ T cell response. It has been known for some time that expansion of the CD4 response is less robust than the CD8 response (Homann et al., 2001). Further, the decline of the antigen-specific effector CD4^+^ T cells after the peak of the response is less steep (90–95% of effector CD8s are lost; compared to 75–80% of effector CD4s) within the 2–3 weeks after the peak of the response (**Figure 1**). After that early contraction, memory CD8^+^ T cells are maintained at a constant level while CD4^+^ T cells decline slowly over time (Homann et al., 2001; Pepper et al., 2011; **Figure 1**). However, the pro-apoptotic molecule Bim is critical to the demise of both populations; the absence of Bim spares roughly 80% of the effector CD8^+^ T cells and >90% of the effector CD4^+^ T cells (Wojciechowski et al., 2006). Interestingly, there are some basic differences in how effector CD4^+^ versus CD8^+^ T cells combat Bim in order to enter the memory compartment. We and others recently showed that IL-7 and IL-15 contribute to survival of effector CD8^+^ T cells by promoting expression of Bcl-2 through STAT5 (Schluns et al., 2000, 2002; Rubinstein et al., 2008; Tripathi et al., 2010). However, we found that neutralization of IL-7 in IL-15-deficient mice did not result in significantly increased contraction of the CD4^+^ T cell response (Tripathi et al., 2010). Further, we found that, in contrast to CD8^+^ T cells, effector CD4^+^ T cells were much more able to tolerate the loss of STAT5 and persisted for some time as STAT5^low^ effector T cells (Tripathi et al., 2010). Similarly, neutralization of Bcl-2 does not exacerbate contraction of the CD4^+^ T cell response (Tripathi et al., 2007), suggesting that, in effector CD4^+^ T cells, something other than Bcl-2 combats Bim. Thus, while there are some similarities with effector to memory transition between CD4^+^ and CD8^+^ T cells more work is necessary to untangle the mechanisms that control this transition.

## FUTURE DIRECTIONS

Recent progress has greatly improved our understanding of how memory T cells emerge from the effector pool. Death and differentiation work together to shape the effector T cell response. Most effector T cells that are generated die shortly after the peak of the response. This death process is largely mediated by the pro-apoptotic Bcl-2 family member, Bim. Bim function is negatively controlled by the levels of Bcl-2, which are regulated by the availability of common gamma chain cytokines. Death and differentiation could be manipulated to enhance the death of autoreactive T cells. On the other hand, manipulation of death and differentiation processes could be exploited to improve vaccine responses. For example, recent work from us and other have suggested that IL-7 may be an excellent vaccine adjuvant, promoting strong effector T cell responses to help B cells make antibody as well as promoting strong effector CD4^+^ and CD8^+^ T cell responses (Tripathi et al., 2007; Nanjappa et al., 2008; Nam et al., 2010; Pellegrini et al., 2011). However, the effects of IL-7 are somewhat short-lived as they wane with the withdrawal of the cytokine. Thus, factors that restrict cellular differentiation (i.e., IL-10, IL-21) may be combined with IL-7 therapy to boost long-lived central memory T cells. This may be particularly advantageous for vaccines that require boosting to achieve immunity, such as the hepatitis B vaccine. Conversely, other vaccines may benefit from effector memory T cells, which are maintained in the tissues and provide substantial protection from tissue borne infections (Bachmann et al., 2005). For example, adenoviral vaccines appear to promote strong effector T cells that appear to persist as effector memory cells (Reyes-Sandoval et al., 2011). Thus, more research is necessary to define successful immunization strategies that maximize protective immunity. Exploitation of combinatorial strategies aimed at controlling the type of inflammation with enhancing effector T cell survival may provide approaches that could be tailored to the particular infectious disease.

## Conflict of Interest Statement

The authors declare that the research was conducted in the absence of any commercial or financial relationships that could be construed as a potential conflict of interest.
